# Development of novel amino-ethyl chitosan hydrogel for the removal of methyl orange azo dye model

**DOI:** 10.1038/s41598-024-51538-1

**Published:** 2024-01-13

**Authors:** Tamer M. Tamer, Rafik Abbas, Wagih A. Sadik, Ahmed M. Omer, Mai M. Abd-Ellatif, Mohamed S. Mohy-Eldin

**Affiliations:** 1 Polymer Materials Research Department, Advanced Technologies and New Materials Research Institute (ATNMRI), City of Scientific Research and Technological Applications (SRTA-City), P.O. Box 21934, New Borg El-Arab City, Alexandria Egypt; 2 Institute of Graduate Studies and Research, Alexandria University, P.O:832, Qesm Bab Sharqi, 21526 Alexandria Egypt

**Keywords:** Environmental sciences, Chemistry, Materials science

## Abstract

The present study introduces a new and straightforward method for the amination of Chitosan. This method involves coupling Chitosan (CS) with 2-chloroethylamine (ENH2) in a single step to produce an amino-ethyl Chitosan derivatives with increased amine group content (CS-ENH2) using click chemistry. The resulting derivatives were then crosslinked using Glutaraldehyde to form amino-ethyl Chitosan Schiff bases. The novel amino-ethyl Chitosan Schiff bases were subsequently utilized as adsorbents for the removal of Methyl Orange (MO) dye from aqueous solutions using a batch technique, and the performance of the produced Schiff bases was compared with that of the native Chitosan Schiff base. The CS-ENH2 adsorbents show improved adsorption capacity up to 300% of the native Chitosan Schiff base with almost double removal rate. The adsorption temperature has a positive impact in general while almost 100% of MO removed at 60 °C using CS-ENH2 adsorbents compared with 66% of the native Chitosan Schiff base adsorbent. The adsorption pH shows a negative impact on the MO removal percent. That effect reduced sharply using the CS-ENH2 adsorbents with higher amination degree while the MO removal percent almost being constant over a wide range of pH; 2.0–7.0. The agitation speed has the same positive effect over all the adsorbents. However, the rate of MO removal percent decreased with increase the agitation speed up to 250 rpm. The experimental findings demonstrated that the highest percentage of MO dye removal was achieved under the conditions of pH 2.0, a temperature of 60 °C, agitation speed of 250 rpm, and adsorption duration of 90 min. These Schiff bases were subsequently characterized using advanced analytical techniques including Fourier Transform Infrared spectroscopy, Thermal analysis (TGA and DSC), and Scanning Electron Microscopy.

## Introduction

Industries that work with dyestuff, textiles, leather, paper, plastics, etc., typically drain synthetic dyes along with their effluent. The synthetic dye wastewater is mainly composed of organic components, which are complex and easy to show color in water, and usually slow or difficult to degrade in the natural environment^1,2^. Among the synthetic dyes, anionic azo dyes account for half of the dye synthesis and industrial application^3^. Due to the low coloring rate on natural fibers, anionic dyes account for a large proportion of the dye wastewater discharged by printing and dyeing factories. Methyl orange [(MO) dimethylaminoazobenzenesulfonate] is a common and typical azo anionic dye. This water-soluble organic synthetic dye has very high colorability and presents a bright orange color when dissolved in water. Azo dyes such as methyl orange contain aromatic and –N=N– groups in their molecules, which are highly toxic, carcinogenic and teratogenic^4,5^, and are harmful to the environment and organisms^6,7^. In addition, the dyes in the wastewater can lead to the deterioration of water quality^8^, so the wastewater containing dyes must be treated innocuously and the dye components need to be removed in order to discharge to the natural water environment or carry out secondary use^9,10^. The adsorption process is considered a valuable technique for the removal of dyes from wastewater, alongside many other physical and chemical processes^11–13^. Numerous research endeavours have been conducted with the aim of identifying cost-effective and efficient adsorbents for the purpose of reducing dye concentrations in aqueous solutions. The researchers incorporated several materials, such as activated carbon, peat, chitin, silica, among others^14^. Chitosan or its derivatives have been demonstrated to be an efficacious substance for the removal of anionic dyes^15–22^ through its amino groups which have the ability to undergo cationization, resulting in a strong electrostatic interaction with anionic dyes when exposed to acidic conditions.

In order to enhance the adsorption efficiency, previous studies have indicated that the adsorbent material underwent modification by the introduction of high chelating coordination of sulphur (S) and nitrogen (N) functional groups^23–26^. Nevertheless, the publications outlined a multitude of reaction steps required for polyamine design. Aminated Chitosan derivatives were synthesized by many authors using different approaches, however these studies used two or even three steps to achieve their goal^23–31^. In a recent study^32^, aminated Chitosan materials have been developed using a one-pot method. Further enhancement of the adsorption capabilities of Chitosan derivatives has been tried through crosslinking process using various cross-linking reagents which have dual purpose namely, stabilizing Chitosan in acid solutions, rendering it insoluble, and augmenting its mechanical qualities.

The novelty of the current study focused on presents a newly developed one-step amination technique for Chitosan through coupling of the Chitosan (CS) with 2-chloroethylamine (ENH2) using click chemistry. The obtained Chitosan derivatives with an increased content of amine groups (CS-ENH2) have subsequently crosslinked using Glutaraldehyde, leading to the formation of amino-ethyl Chitosan Schiff bases. The CS-ENH2 Schiff bases were used as an adsorbent to remove Methyl Orange (MO) dye from aqueous solutions in batch mode process, and its performance was compared with the native Chitosan Schiff base. The impact of several factors, including adsorption time, initial dye concentration, adsorption temperature, adsorption pH, agitation speed, and adsorbent dosage, on the adsorption of Mo dye have been studied. These Schiff bases are further characterized using advanced analytical techniques such as Fourier Transform Infrared spectroscopy (FTIR), Thermal (TGA and DSC) analysis, and Scanning Electron Microscopy (SEM).

## Materials and methods

### Materials

Chitosan medium molecular weight (≥ 75% DD) and methyl orange dye (MO; 85%) were procured from Sigma-Aldrich (Germany). The compound 2-Chloro ethyl amine hydrochloride (99%) was acquired from Sigma-Aldrich (Germany). The other chemicals used in this study were obtained from El-Nasr Pharmaceutical Co for Chemicals (Egypt) included sulfuric acid (98%), sodium hydroxide (99%), and phenolphthalein (98%). The Glutaraldehyde (GA) used in this study was a 25.0 wt% aqueous solution acquired from ACROS Organics.

### Methods

#### Preparation of amino-ethyl chitosan hydrogel

A solution containing 2 grammes of CS was prepared by dissolving it in 40 millilitres of acetic acid with a concentration of 2%. Subsequently, different quantities of Cl-ENH2 (0, 6.4, 12.7, 25.5, and 51 mM) were added individually to the Chitosan solution and the obtained adsorbents were coded as CS, CS-ENH2-1, CS-ENH2-2, CS-ENH2-3, and CS-ENH2-4, respectively. The temperature was then increased to 70 degrees Celsius and the mixture was stirred for duration of 2 h. Subsequently, a volume of 0.5 ml of Glutaraldehyde solution with a concentration of 25% was introduced into the mixture while maintaining a consistent stirring motion for duration of one hour. The CS-ENH2 hydrogel was subjected to drying at a temperature of 60 °C for duration of one night. The dried samples underwent a milling process and were subsequently subjected to multiple washes using hot distilled water in order to eliminate any remaining unreacted components. Subsequently, the samples were subjected to a drying process and subsequently stored within desiccators to facilitate subsequent analysis and adsorption tests.

#### Structural and morphological Characterization

##### Infrared Spectrophotometric (FTIR)

To confirm the change and establish the structure of the hydrogel, Fourier transform infrared spectroscopy was conducted using a Shimadzu FTIR-8400 S spectrophotometer from Japan.

##### Thermal gravimetric analysis (TGA)

The analysis of materials was conducted using a thermo gravimetric analyzer (Shimadzu TGA –50, Japan) with a Nitrogen flow rate of 30 ml/min. This analysis aimed to observe any structural changes resulting from the modification. The measurement of weight loss in the samples commenced at room temperature and continued up to 600 °C, with a heating rate of 10 °C per minute.

##### Differential scanning calorimeter (DSC)

Differential scanning calorimetry was performed on the samples using a Shimadzu DSC-60A apparatus from Japan. The analysis was conducted over a temperature range from ambient to 350 °C, with a heating rate of 10 °C/min and under a nitrogen flow of 30 ml/min.

##### Scanning electron microscopic analysis (SEM)

Prior to examination by scanning electron microscopy, the samples were subjected to a vacuum environment and coated with a thin layer of gold. The morphological changes on the surface of the samples were monitored using a secondary electron detector of a scanning electron microscope (SEM) model Joel Jsm 6360LA, manufactured in Japan.

#### Physicochemical characterization

##### Water uptake (%)

The Water uptake behaviour of the prepared hydrogel was investigated using distilled water (pH 5.4). Accurately weighed amounts of hydrogels were immersed in water and allowed to swell for 24 h at R.T. The swollen hydrogel was periodically separated, and the moisture adhered to the surface of hydrogel was removed by blotting them gently in between two filter papers, immediately followed by weighing. The swelling degree of samples was determined according to the following formula^33^:

1 $${\text{Water uptake }}\left( \% \right) \, = \, \left[ {\left( {{\text{M}}_{{\text{t}}} - {\text{M}}_{0} } \right) \, /{\text{ M}}0} \right] \, \times {1}00$$

where M_t_ is the weight of the swollen hydrogel, and M0 is the initial dry weight.

##### The ion exchange capacity

A known weight of chitosan or schiff base hydrogels were added to the known volume of 0.1 M H_2_SO_4_ solution, and the mixture was kept under shaking for three h. The mixture was filtered, and an aliquot was titrated against a standard solution of sodium hydroxide. Similarly, control titration without the addition of Chitosan was also run. From the difference in the volume of NaOH required for neutralization, the ionic capacity of chitosan samples was calculated using the following equation:

2 $${\text{Ion exchange capacity }} = \, \left( {{\text{V}}_{{2}} - {\text{V}}_{{1}} } \right){\text{ A }}/{\text{ W }}\left( {{\text{meq}}/{\text{g}}} \right)$$

where V_2_ and V_1_ are the volumes of NaOH required for complete neutralization of H_2_SO_4_ in the absence and presence of chitosan membrane, respectively, A is the normality of NaOH and W is the weight of sample taken for analysis^34^.

#### Batch equilibrium studies

A stock solution of methyl orange (MO) dye with a concentration of 1g/L (1000 ppm) was made in distilled water. Subsequently, the desired concentrations, 10, 20, 25, 50, and 100 ppm, were achieved by diluting of 1, 2, 2.5, 5, and 10 mL of the stock solution with distilled water up to 100 mL using 100 mL flasks. The adsorption tests were performed using 100 mL flasks. A certain quantity of adsorbent was added to 25 mL dye solution with varying dye concentrations and pH values. The mixture was then agitated in an orbital shaker for a predetermined duration. The concentrations of MO in the initial and final aqueous solutions were determined by employing a UV–Vis spectrophotometer set to a wavelength of 465 nm. The quantity of dye adsorbed was determined by subtracting the initial concentration from the equilibrium concentration. The calculation of the percentage elimination value was determined using the following mathematical relationship:

3 $${\text{Dye removing }}\left( \% \right) \, = \, \left( {{\text{C}}_{0} - {\text{C}}_{{\text{t}}} } \right)/{\text{C}}_{0} \times {1}00$$

where; C_0_ is the initial dye concentration and C_t_ is the final dye concentration in supernatant.

## Results and discussion

### Characterization

#### Physicochemical characterization

**Figure 1:**
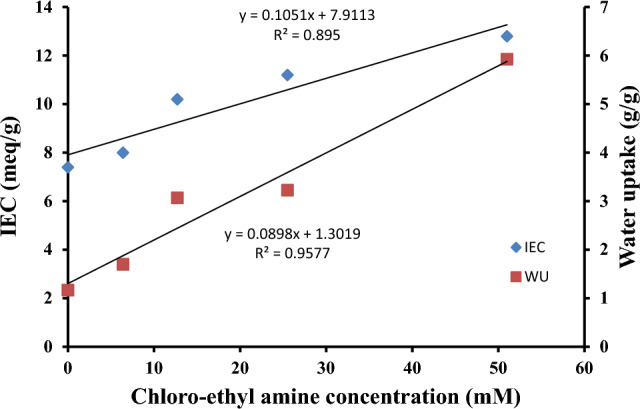
Water uptake and ion exchange capacity of Chitosan and amino-ethyl Chitosan hydrogels.

The confirmation of the immobilisation of excess amine into the chitosan backbone is achieved through the measurement of ion exchange capacity. Figure 1 illustrates a nearly linear augmentation in the ion exchange capacity as the quantity of reacted 2-chloroethyl amine in the modification procedure is elevated. This finding substantiates the observed elevation in the concentration of free amines inside the hydrogels that were synthesised.

The water absorption capacity of the hydrogels that were created was assessed and is illustrated in Fig. 1. A linear rise in water absorption is seen, providing confirmation of the immobilisation of more free amino groups. Furthermore, the incorporation of immobilised ethyl amine groups as grafted chains onto the Chitosan backbone serves several purposes. Firstly, it enhances the internal space within the hydrogel. Secondly, it reduces the crystallinity of the hydrogel. Lastly, it increases the size of the three-dimensional network, thereby promoting the diffusion of water molecules and consequently augmenting the amount of water uptake.

#### Infrared spectrophotometric

**Figure 2:**
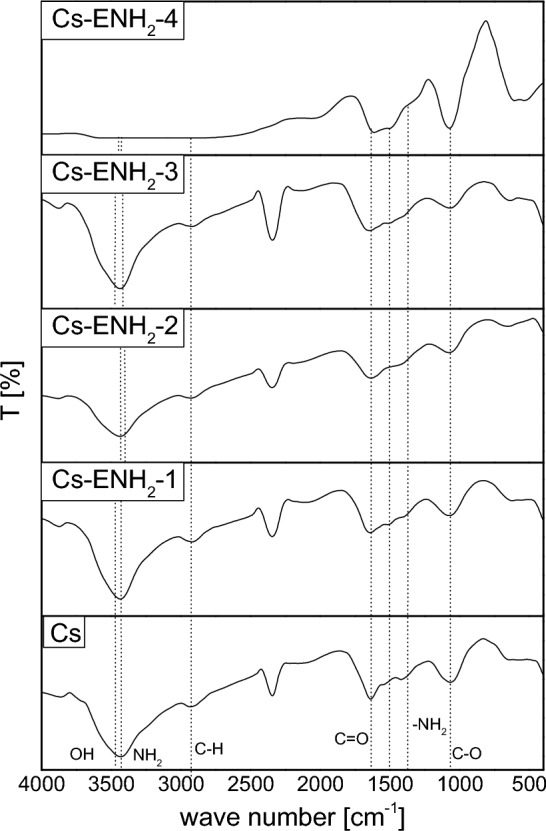
FTIR of Chitosan and amino-ethyl Chitosan hydrogels.

The characteristics’ spectrum of CS displays a strong absorption band at 3437 cm^−1^ due to OH and amine N–H symmetrical stretching vibration. A peak at 2921 cm^−1^ was due to symmetrical C-H stretching vibration attributed to pyranose ring. The sharp peak at 1383 cm^−1^ was assigned to CH3 in amide group. The broad peak at 1095 cm^−1^ was indicated C–O–C stretching vibration in CS^30^, peaks at 1649 and 1425 cm^−1^ were due to C=O stretching (amide I) and N–H stretching (amide II). The absorption band at 1153 cm^-1^ was assigned to the anti-symmetric stretching of C–O–C bridge and 1095 cm^−1^, 1010 cm^−1^ were assigned to the skeletal vibration involving the C–O stretching. In the other hand, the infrared spectrum of ethylamine, wave numbers ~ 1500 to 400 cm^−1^ is considered the fingerprint region for the identification of ethylamine and most organic compounds. It is due to a unique set of complex overlapping vibrations of the atoms of the molecule of ethylamine. The most prominent infrared absorption lines of ethylamine at wavenumbers ~ 3500 to 3300 cm^−1^ is a broad band for N–H bond stretching vibrations, characteristic of amines. The hydrogen bonding interferes with the N–H stretching vibrations producing the broad band peaking at around ~ 3400 cm^−1^. There are also characteristic bands due to N–H vibrations at wave numbers 1650–1580 cm^−1^. Vibrations characteristic of C-N bonds in aliphatic amines like ethylamine occur at 1220–1020 cm^−1^. Around 3000–2800 cm^−1^ are absorptions due to C–H stretching vibrations—they overlap with the N–H stretching vibrations. The similarity between most of the characteristic peaks of the Chitosan and 2-Chlorethyl amine, makes overlapping of both parent materials peaks. Figure 2 depict the Fourier-transform infrared (FT-IR) spectroscopic analysis of Chitosan and amino-ethyl Chitosan hydrogels. The provided charts depict the characteristic bands of functional groups in polysaccharides, including prominent band within the range of 3400 cm^−1^, which correspond to the vibrational modes of hydroxyl and amine groups involved in starching. Chitosan shows band at a wave number of 3433.4 cm^−1^. A shift of the wave number to 3435.33, 3439.2, 3437.26, and 3338.9 cm^−1^ of the CS-ENH2-1, CS-ENH2-2, CS-ENH2-3, and CS-ENH2-4, respectively, has been recognized with varied absorption intestines. The presence of bands at a wave number of 2938 cm^−1^ is indicative of the presence of aliphatic C–H bonds. A shift of the wave number to 2924, 2930, 2922, and 3014.84 cm^−1^ of the CS-ENH2-1, CS-ENH2-2, CS-ENH2-3, and CS-ENH2-4, respectively, has been recognized with varied absorption intestines. The amide groups of Chitosan were seen at a wave number of 1641 cm^−1^. A shift of the wave number to 1637.62, 1653, and 1614.47 cm^−1^ of the CS-ENH2-2, CS-ENH2-3, and CS-ENH2-4, respectively, has been recognized with varied absorption intestines. The spectral bands observed within the range of 1000–1300 cm^−1^ are associated with the C–O stretching vibrations occurring in the glucose ring of Chitosan. Chitosan shows band at a wave number of 1068.6 cm^−1^. A shift of the wave number to 1076.3, 1078.24, 1070.53, and 1074.39 cm^−1^ of the CS-ENH2-1, CS-ENH2-2, CS-ENH2-3, and CS-ENH2-4, respectively, has been recognized with varied absorption intestines.

#### Thermal gravimetric analysis (TGA)

**Figure 3:**
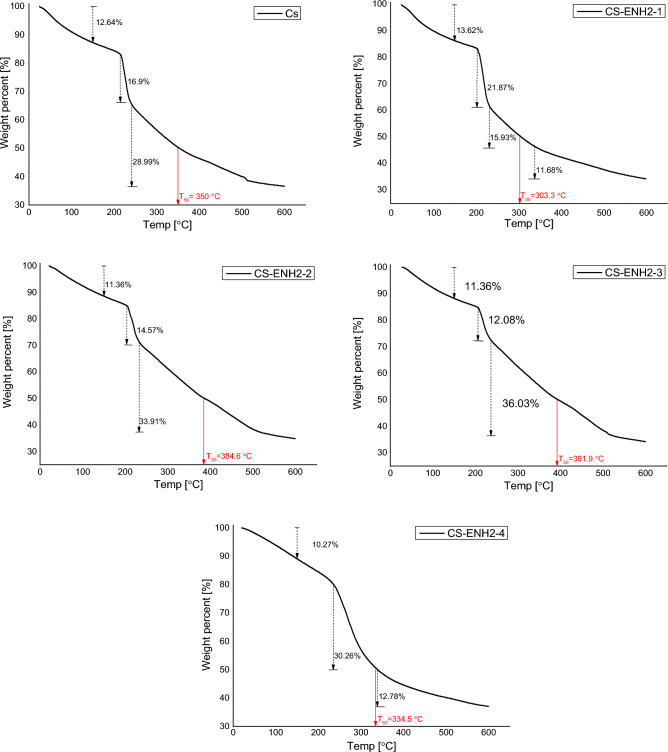
TGA of Chitosan and amino-ethyl Chitosan hydrogels.

Thermogravimetric analysis (TGA) was conducted on the hydrogel derivatives of CS and CS-ENH2, and the results are illustrated in Fig. 3. The presented chart displays data pertaining to the thermal degradation process occurring in the presence of a Nitrogen atmosphere. The observed trend indicates a gradual decline in the measured weight samples values, commencing from the initial temperature of the surrounding environment and continuing until about 150 °C. The recorded samples weight loss percent values fall within the range of 10.87–12.79%, which can potentially be attributed to the reduction in moisture content inside the polymers, as suggested by previous studies. The occurrence of a secondary samples weight loss percent at an elevated temperature, ranging from 17.48 to 27.25%, can perhaps be attributed to the oxidative degradation of the pyranose ring within the chitosan backbone. During this phase, the occurrence of samples weight loss can be attributed to the decomposition of the amine groups inside the pyranose ring, leading to the formation of novel crosslinked fragments. The residue that was generated underwent a gradual decomposition process by the application of increased temperature within the range^35–37^.

#### Differential scanning calorimetry (DSC)

**Figure 4:**
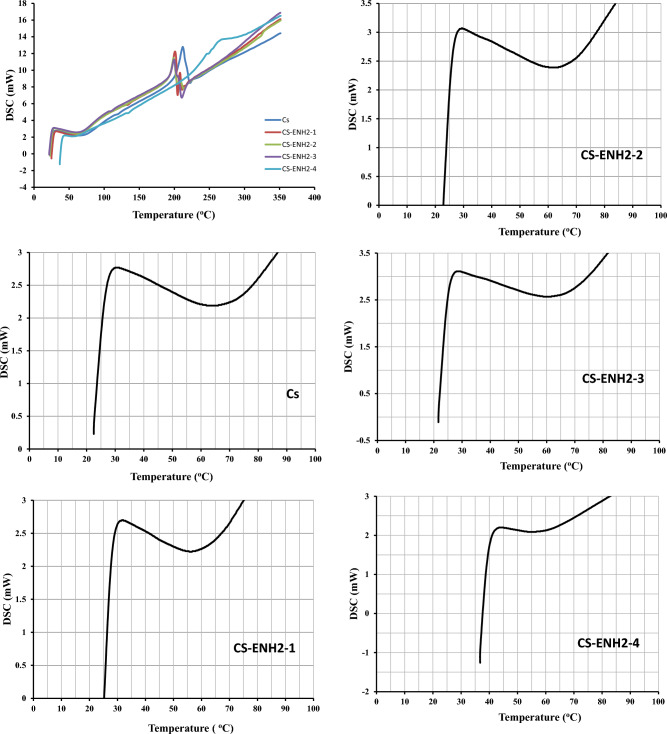
DSC of Chitosan and amino-ethyl Chitosan hydrogels.

The differential scanning calorimetry (DSC) investigation was performed on the hydrogel derivatives of CS and CS-ENH2, as depicted in Fig. 4. The initial endothermic peak observed in all studied materials, occurring between 50 and 120 °C, can be attributed to the increase in moisture content. The inclusion of hydrophilic functional groups, such as hydroxyl and amine groups, along the polymer chain imparts a strong attraction to water molecules. This property allows the polymer to effectively absorb and retain water from the surrounding atmosphere or during the production process. The second thermal event observed in the chart can perhaps be attributed to the disintegration of the glucose amine (GlcN) units inside the Chitosan hydrogels. This decomposition process is characterised by an exothermic peak occurring at a temperature of 210 °C^38^.

#### Scanning electron microscope (SEM) and EDAX analysis

**Figure 5:**
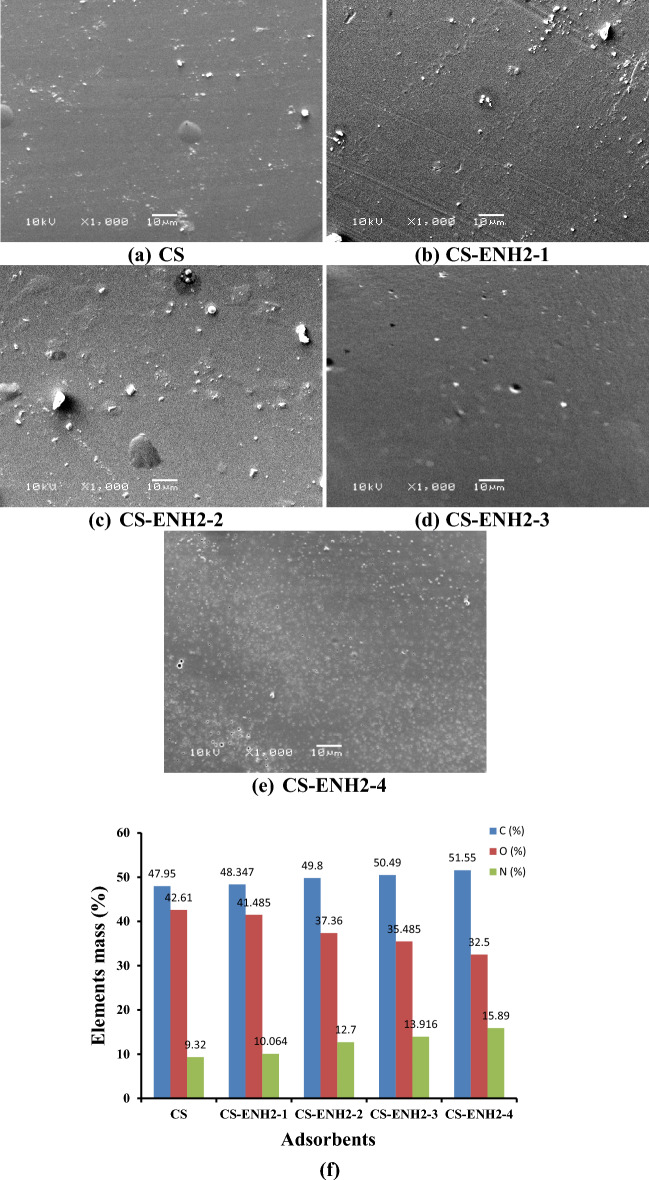

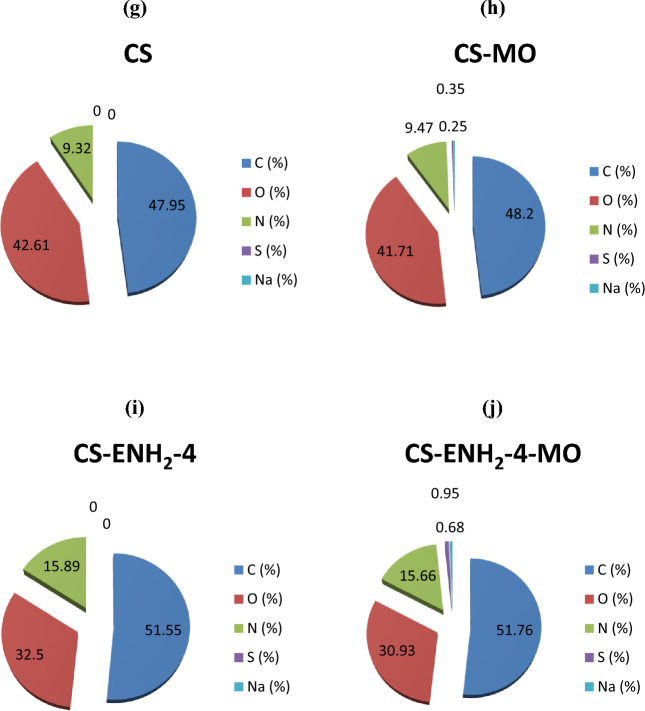
SEM pictures of Chitosan and amino-ethyl Chitosan hydrogels (**a**–**e**), and EDAX analysis of (**f**) Chitosan and amino-ethyl chitosan derivatives, (**g**) CS, (**h**) CS-MO, (**i**) CS-ENH2-4, and (**j**) CS-ENH2-4-MO.

The morphological study of Chitosan and amino-ethyl Chitosan hydrogel was conducted utilising a scanning electron microscope, as depicted in Fig. 5a–e. The images demonstrate a marginal elevation in surface roughness and pore structure as the amine content of chitosan is augmented. The observed phenomenon can be attributed to the favourable interaction between the extended side chains containing terminal amine groups and the molecular structure of Chitosan. These chains offer an alternative active site for the process of crosslinking, as opposed to the original amine groups, perhaps resulting in increased space between polymer chains. The verification of the structural modifications of Chitosan to amino-ethyl Chitosan by introducing ethyl amine groups has been conducted using EDAX analysis (Fig. 5f). The study reveals an increase in the mass percentage of carbon (C) and nitrogen (N) in comparison to the oxygen (O) mass percentage observed in the Chitosan blank sample. Figure 5g, h present empirical support for the adsorption of MO on the Chitosan adsorbent, as indicated by the presence of S and Na elements and an increase in the mass percentage of C and N, accompanied by a corresponding decrease in the mass percentage of O. On the contrary, the adsorption of MO on the CS-ENH2-4 adsorbent (Fig. 5j) exhibits an increase in the presence of S and Na elements compared to the CS-ENH2-4 adsorbent (Fig. 5i). Additionally, there is a slight increase in the C mass percent, while the N and O mass percents experience a slight decrease. It is noteworthy to mention that the elements S and Na exhibit a higher mass percentage in comparison to Chitosan-MO, as depicted in Fig. 5h. This observation provides evidence for the enhanced performance of the CS-ENH2-4 adsorbent in comparison to Chitosan.

### Adsorption process

This study explores the sorption characteristics of methyl orange (MO) using Chitosan and amino-ethyl Chitosan hydrogels in the presence of artificially contaminated water solution. A series of experiments were conducted to examine the adsorption characteristics of hydrogels towards MO under various adsorption settings, including adsorption time, adsorption temperature, adsorption pH, agitation rate, adsorbent dosage, and starting dye concentration.

#### Effect of the adsorption time

**Figure 6:**
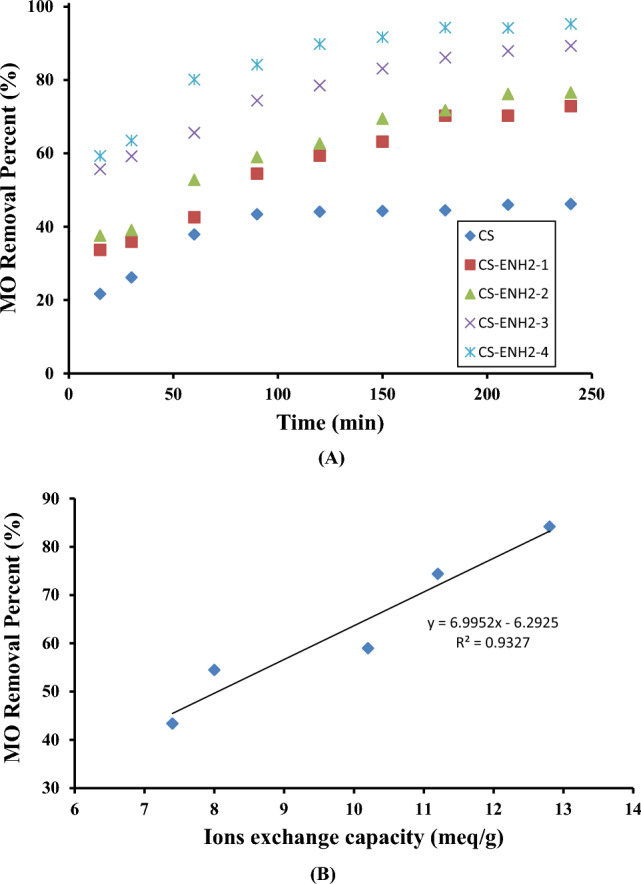
(**A**) Effect of contact time of adsorption behavior of MO onto Chitosan and amino-ethyl Chitosan hydrogels. (**B**) Correlation between ions exchange capacity of Chitosan and amino-ethyl Chitosan hydrogels and MO removing (%).

Figure 6a depicts the impact of adsorption time on the efficacy of MO removal percentage (%) using different Chitosan adsorbents. From the Figure, It is acknowledged that the percentage of dye removal exhibits a linear rapid progress as a first initial period for all the Chitosan adsorbents. That period varied with the amine content of the adsorbent. For Chitosan, it is recognized to be 90 min, which after almost no noticeable MO removal was noticed suggesting the consumption of all the amine active adsorption sites and attainment of equilibrium. In the other hand, it can be noticed that all the derivatives of amino-ethyl Chitosan exhibit a higher linear increase in the percentage of MO removal, compared to Chitosan, in the following order CS-ENH2-1, CS-ENH2-2, CS-ENH2-3, and CS-ENH2-4 correlated to the increment of the amine content adsorption sites. At this stage, all the active adsorption sites are free and accessable to MO molecules, in addition to the existence of high MO concentration gradient between the adsorbents solid phase and the MO liquid phase. These driving force leads to a high rate of MO removal compared to Chitosan counterpart. Subsequently, with a progress of adsorption time, that driving force was reduced as a result of consuming most of the adsorption active sites and reduced of the concentration gradient. A slower rate of increase is observed until reaching saturation at 240 min where the adsorption is controlled by the diffusion of MO molecules to the interior pores of the adsorbents. The performance of Chitosan is influenced by secondary key factor namely hydrophobic interactions between the hydrophobic aliphatic moieties of the adsorbents and the aromatic ones of the MO molecules. The hydrophobic interactions arise from the presence of the methyl group in the acetamide moiety (inside partial acetyl amine groups) and the –CH and –CH2 groups in the glucose ring. The adsorption process of MO, an anionic dye, onto Chitosan adsorbents was conducted using a combination of physical sorption behavior and chemisorptions, which occurred due to the electrostatic interaction between opposite charges in accordance with previously published data where Chitosan Schiff bases have used in the removal of MO dye from aqueous solution^39–41^. The Chitosan structure as a linear cationic polymer was modifie to improve its behaviour via preparing two different crosslinked Chitosan Schiff bases hydrogels using a glutaraldehyde crosslinker. The Chitosan was coupled with succinimide (Ch/Su) and 1-methyl-2-pyrrolidinone (Ch/Mp)^39^. Cross-linked Chitosan derivate Schiff bases obtained from the coupling of Chitosan with 1-vinyl 2- pyrrolidone [Schiff base (I)] and 4-amino acetanilide (Schiff base (II))^40^. Other Chitosan Schiff base derivative was developed by reaction of Chitosan with 4-methoxybenzaldehyde to have Chitosan Schiff base (Cs/MeB) in the presence of Glutaraldehyde as a crosslinker^41^.

In the case of the existing adsorbents, augmenting the degree of ethylamine substitution leads to heightened hydrophobic interactions and more advantageous active sites comprising amine groups. In order to investigate the effects of incorporating additional amine groups by fictionalization with 2-chloroethyl amine, the cationic exchange capacity of the amino-ethyl Chitosan derivatives that were synthesized was determined. This measurement was then associated with the percentage of MO removal, as shown in Fig. 6b. The figure illustrates a notable linear correlation between the removal percentage of MO in CS and the concentration of CS-ENH2-4 in the barrel, as seen by the nearly twofold rise in removal percentage from 43.3 to 84.2% when the ion exchange capacity (IEC) of CS and CS-ENH2-4 increased from 7.4 to 12.8 meq/g. This accomplishment demonstrates the efficacy of using more amine groups to boost the removal effectiveness of MO, on one hand. On the contrary, this provides an indication of the prevailing electrostatic interaction between opposing charges, specifically the positive charge on CS-ENH2 derivatives and the negative charge on the MO molecules.

#### Effect of temperature

**Figure 7:**
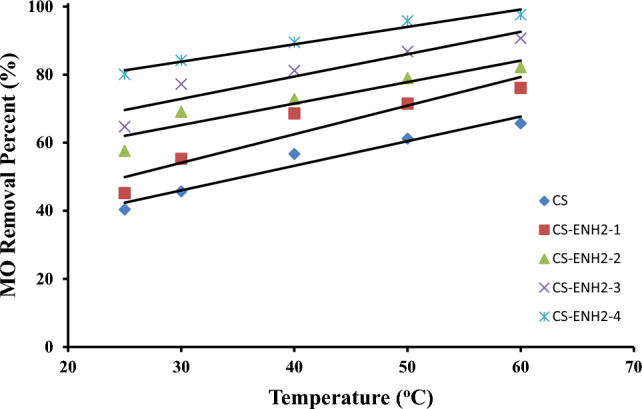
Effect of the adsorption temperature on the MO removing (%) using Chitosan and amino-ethyl Chitosan hydrogels.

The study investigated the impact of variations in environmental temperature on the percentage of MO removal utilising cross-linked Chitosan and amino-ethyl Chitosan hydrogels. The temperature range examined spanned from 25 to 60 degrees Celsius, as depicted in Fig. 7. Based on the data presented in the Figure, it is evident that there is a consistent linear increase in the percentage of MO removal by all of the adsorbents utilised, with a similar rate of growth observed. The elevation of the medium temperature amplifies the mobility of the big dye ions, so expediting their stochastic motion within the solution. Consequently, this augmentation leads to an increase in the frequency of collisions between the dye ions and the adsorbent surface. Furthermore, it induces an increase in volume inside the internal framework of the adsorbent, hence facilitating deeper penetration of the larger dye molecules^42^. Similar finding where Cross-linked Chitosan derivate Schiff bases obtained from the coupling of Chitosan with 1-vinyl 2- pyrrolidone [Schiff base (I)] and 4-amino acetanilide (Schiff base (II)) were used in the removal of MO dye^40^. The authors explained the effect of the temperature increment would increase the mobility of the large dye ions as well as produce a swelling effect on the internal structure of the Chitosan. That consequently facilitates the diffusion of the large dye molecules^40^. It is noteworthy to notice that the amino-ethyl Chitosan derivatives exhibited a larger boost in the percentage of MO removal at the lowest temperature (25 °C), with the CS-ENH2-4 sample achieving a 100% removal rate. On the contrary, it was observed that the increase in the elimination percentage of MO was only 48.5% at the greatest temperature (60 °C). Hence, the adsorption capacity is predominantly influenced by the chemical interaction occurring between the functional groups present on the internal surface of the adsorbent (namely, the surface of its pores) and the adsorbate. This capacity is expected to augment with increasing temperature.

#### Effect of pH

**Figure 8:**
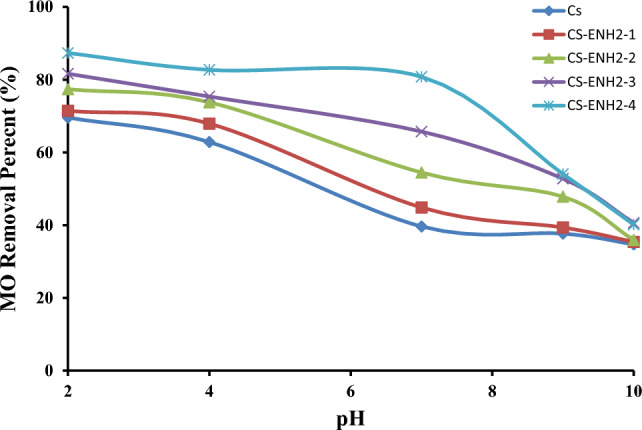
Effect of the dye pH on the adsorption of MO onto Chitosan and amino-ethyl Chitosan hydrogels.

Figure 8 illustrates the impact of the pH value of the initial MO solutions on the efficiency of adsorption. The absorption of MO dye is significantly higher in acidic solutions compared to alkaline settings. Under acidic conditions, the presence of free amine groups along the chitosan backbone leads to their protonation, resulting in the formation of a positive charge on the surface of the hydrogel. This positive charge facilitates electrostatic interactions with the negatively charged sulfonate group of MO. Comparable findings have reported by other authors^43,44^. The adsorption capabilities of chitosan hydrogels are observed to decrease at alkaline pH levels. At the given pH, the surface charges of chitosan exhibited a negative polarity, hence impeding the adsorption process due to the electrostatic repulsion between the negatively charged dye molecules and the adsorbent (chitosan hydrogel). One intriguing finding in this study is the inverse relationship between the removal percentages of MO and the cation exchange capacity of amino-ethyl Chitosan samples. Notably, the CS-ENH2-4 sample exhibited the lowest reduction rate, with a rapid decrease in MO removal % found after reaching a pH of 7.0. On the contrary, it was observed that all the adsorbents exhibited similar percentages of MO removal in pH 10.0, ranging from 35 to 40%. This observation shows that the primary factor influencing the adsorption process is the hydrophobic-hydrophobic interaction^45^. Similar finding results where joint steady adsorption pH range from 6.0 to 8.0 of the Chitosan Schiff bases adsorbents can be explained by two reasons. The first is the reduced number of the free amine groups’ numbers affected by the deprotonation. The second is to increase the physical adsorption role of the chitosan Schiff bases adsorbents via hydrophobic-hydrophobic interaction, which is expected to be higher in the Ch/Mp Schiff base hydrogel containing heterocyclic ring with an attached methyl group^39^. On the other hand, the dye removal % by Cs/MeB was slightly affected by the increase of pH were decreased from 95% at pH 4.0 to 86% at pH 9.0. This behaviour confirmed the dominated hydrophobic-hydrophobic physical adsorption between the benzene rings and the methyl hydrophobic groups of the Cs/MeB adsorbent and the MO dye molecules in a wide range of pH; from 4.0 to 9.0, while the elimination of the cationic charge of the last free amine groups at pH 10.0 leads to the collapse of the Cs/MeB hydrogel structure with loss of its water content leading to reduce the pores volume and so the internal pores surface area. The high and almost constant MO removal % by Cs/MeB hydrogel in a wide pH range is a great advantage for its application in the treatment of industrial effluents contaminated with MO dye^41^.

#### Effect of agitation rate

**Figure 9:**
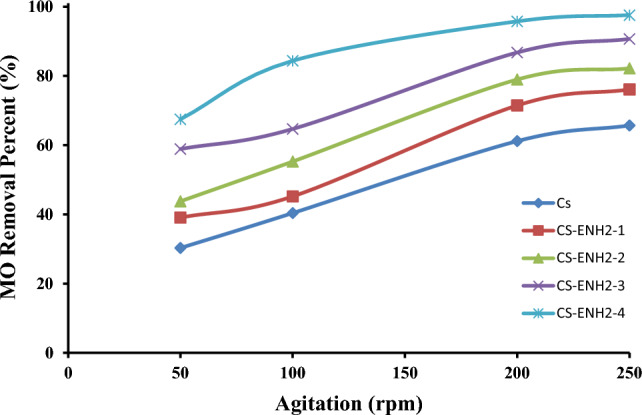
Effect of the agitation rate on the adsorption of MO onto Chitosan and amino-ethyl Chitosan hydrogels.

The impact of agitation rate on the adsorption of MO was investigated by conducting experiments at various agitation rates ranging from 50 to 250 rpm, while keeping the kinetic parameters constant. Figure 9 presents an overview of the findings derived from the study. The adsorption of the MO is observed to exhibit a notable enhancement as the agitation rate is raised within the range of 50–200 rpm, after which it reaches a plateau until 250 rpm. These findings can be attributed to the observation that higher agitation speeds enhance the diffusion of MO towards the surface of the adsorbents. The relationship between intraparticle diffusivity and adsorption capacity, as well as the surface characteristics of adsorbents, has been suggested to be of significant importance. Increasing the agitation rate has the potential to surpass the thickness of the liquid layer and the resistance to mass transfer on the surfaces of the adsorbents being examined. Therefore, it can be inferred that a shaking rate of 200 rpm is adequate to facilitate the accessibility of all surface binding sites for the uptake of methyl orange^40,46,47^.

#### Effect of the initial dye concentration

**Figure 10:**
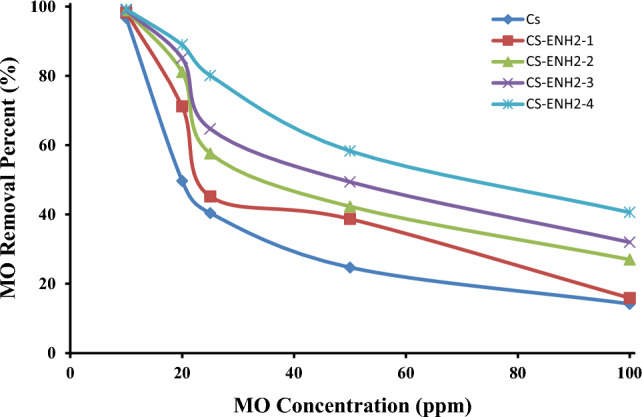
Effect of the initial MO dye concentration on the adsorption of MO using Chitosan and amino-ethyl Chitosan hydrogels.

The impact of the initial concentration of methyl orange on the process of adsorption was investigated over a range of concentrations (10, 20, 25, 50, and 100 ppm). The findings of this investigation are presented in Fig. 10. The figure yields two primary observations. The initial observation pertains to the complete elimination of the lowest concentration (10 ppm) of MO by all hydrogel samples employed. This outcome can be attributed to the presence of an ample number of active sites on the Chitosan sample, thereby accommodating all MO molecules. Consequently, the aminated chitosan samples, which possess a greater number of active sites, do not exhibit any discernible impact due to the limited availability of MO. On the other hand, a notable increase in the percentage of removal of MO has been seen as the initial concentration of MO is increased up to 100 ppm. Specifically, the CS-ENH2-4 sample exhibits a four-fold higher removal percentage of MO compared to the CS sample. The second primary observation pertained to the decline in the percentages of MO removal as the starting MO concentration increased. The process of reduction consists of two distinct steps. The initial acute stage was noticed when the concentration of MO was increased from 10 to 25 ppm. This resulted in a decrease in the reduction percentage, in conjunction with the degree of amination of the aminated chitosan samples. Eventually, a nearly linear reduction rate was achieved with the CS-NH2-4 sample. The second stage of reduction, characterised by a nearly identical decrease in the rate, was observed for all samples of adsorbents, with a recognition threshold of up to 100 ppm. A similar finding was observed where the removal percentage linearly reduced with an increase in initial MO concentration using 4-dimethylamino benzaldehyde chitosan Schiff base and benzophenone chitosan Schiff base. This trend is due to the electrostatic repulsion between the dye molecules with increasing concentration, resulting in a competition between the dye molecules for the limited active sites in the adsorbent^48^.

#### Effect of the absorbent dose

**Figure 11:**
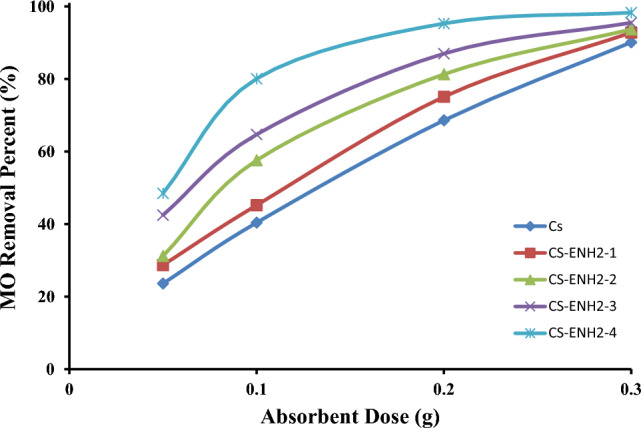
Effect of the adsorbent dose on the adsorption of the MO using Chitosan and amino-ethyl Chitosan hydrogels.

Figure 11 illustrates the impact of varying doses of Chitosan adsorbents on the percentage of MO removal, while keeping the kinetic parameters constant. In general, it was observed that an escalation in the dosage resulted in a proportional rise in the elimination % of MO for both CS and CS-NH2-1 samples, exhibiting a nearly linear relationship. The clearance percentages of MO exhibited a gradual increase when lower rates were applied to the other aminated samples. Ultimately, the adsorption efficiency achieved with 0.3 g of adsorbents exhibits minimal variation, falling within the range of 90–100%. The CS-NH2-4 sample has a plateau effect, commencing at a dosage of 0.2 g, whereby it achieves a clearance percentage of 95%. This phenomenon may be attributed to the observation that, when the starting dye concentration remains constant, increases in the quantity of adsorbent material results in a larger surface area and a greater number of sorption sites^49,50^. Similar quite tendency have been reported using other sorbents reported in the previous work^51^.

#### Reusability

**Figure 12:**
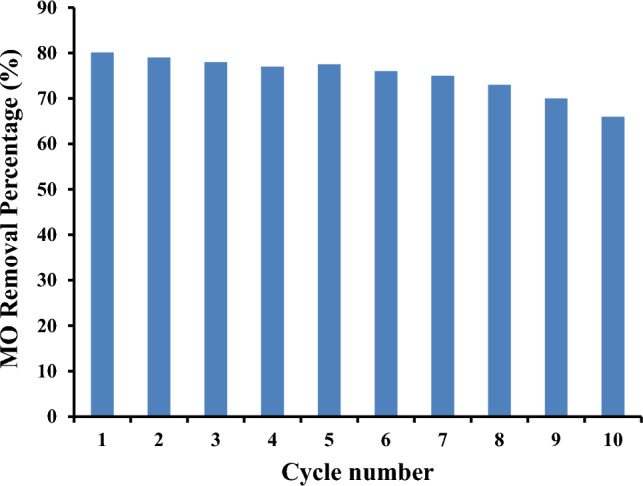
Reusability of the CS-NH2-4 adsorbent in removal of MO dye; [Adsorption conditions; 25 ppm MO, 60 min, pH 7.0, 25 °C, and 0.1 g adsorbent. Desorption conditions; 0.1N NaOH, 25 °C, 60 min].

The sorption–desorption cycle, as depicted in Fig. 12, can be utilised to estimate the recovery of MO absorbed from aqueous solution. The reusability of the CS-NH2-4 adsorbent for removing MO from aqueous solutions was investigated by analysing the sorption–desorption cycles. The cycle was repeated ten times using a sodium hydroxide solution. The figure clearly demonstrates a continuous, nearly linear reduction in MO removal. However, the decrease in MO removal efficiency was not significant; the percentage of MO removal reached 66% in the tenth cycle, compared to 80.1% in the first cycle. Only 18% of the MO removal efficiency was lost after ten cycles of sorption–desorption processes. The CS-NH2-4 adsorbent exhibits favourable sorption–desorption performance and can be confidently used without a noticeable decrease in its sorption capacity for MO removal.

#### Comparative adsorption capacity study

Table 1 presents a comparison of the highest adsorption capacity for MO on the CS-NH2-4 adsorbent in relation to other adsorbents documented in the literature^39–41,48,52–55^. Based on the tabular data, it can be observed that the adsorption capacity of the hydrogels composed of alginate and alginate/poly aspartate is comparatively lower than that of the hydrogels being analysed^55^. The example experienced a reversal upon the utilisation of Calcium alginate MWNTs^53^, resulting in a six-fold increase in adsorption capacity. In contrast, the adsorption capacity of CS-NH2-4 is comparatively lower when compared to other Chitosan and Chitosan derivatives^39–41,52^. Magnetic multi-walled carbon nanotubes (MWCNTs) and bottom ash have also demonstrated elevated adsorption capacity^53,56^. The modest adsorption capacity of the CS-NH2-4 adsorbent can be attributed to several factors, which can be summarised as follows:

1. The scarcity of amine active sites that are protonated for the purpose of adsorption at a pH level of 7.0.
2. The limited expansion of the material at a pH level of 7.0, resulting in a diffusion constraint for the MO (material of interest).
3. The utilisation of the initially available active amine group sites during chemical crosslinking procedures involving Glutaraldehyde. It is advisable to conduct a more comprehensive investigation into the specific parameters of the crosslinking process in order to enhance the adsorption capacity.

**Table 1:** Comparison of the Adsorption Capacity of CS-NH2-4 adsorbent with Other Adsorbents.

Adsorbent	Adsorption capacity (mg/g)	References
Chitosan—1-vinyl 2-pyrrolidone Schiff base	20	^40^
Chitosan/organic rectorite	5.56	^52^
Calcium alginate MWNTs	12.5	^53^
Magnetic MWCNTs	10.89	^53^
Alginate/polyaspartate hydrogels	0.22–0.28	^55^
Alginate	0.08–0.28	^55^
Bottom ash	3.618	^56^
Chitosan	5.54	^41^
Chitosan/4-methoxybenzaldehyde Schiff base	7.73	
Chitosan	8.867	^39^
Chitosan/succinimide Schiff base	10.0	
Chitosan/1-methyl-2-pyrrolidinone Schiff base	7.2	
Chitosan	19.92	^48^
Chitosan/4-dimethylamino benzaldehyde Schiff base	19.34	
Chitosan/benzophenone Schiff base	22.37	
Chitosan	2.368	This study
Amino Ethyl Chitosan Hydogel (CS-NH2-4)	5.0	

## Conclusion

Novel amino-ethyl Chitosan derivatives (CS-ENH2) with varied and higher amine group content than Chitosan have been developed using one step click chemistry reaction of Chitosan (CS) with 2-chloroethylamine (ENH2) amination reagent, then crosslinked using Glutaraldehyde to form new amino-ethyl Chitosan Schiff bases. The introduced amine groups increased the amine content of Chitosan by 70% which corresponding to increase the cationic exchange capacity of Chitosan (CS) from 7.4 to 12.8 meq/g of CS-ENH2-4 sample. The developed CS-ENH2-4 adsorbent shown 300% adsorption capacity of Methyl Orange (MO) dye solution, 100 ppm, compared to native Chitosan one. The study of adsorption time show fast and linear rate in the first 60 min, then lower rate of adsorption was observed until equilibrium starts to reach at 90 min. The CS-ENH2-4 adsorbent shows an almost constant MO removal percentage over a pH range from 2.0 to 7.0 compared with linear decline of the Chitosan counterpart. Both of the adsorption temperature and agitation speed have the same trend which the MO removal percentage increased along with. The experimental findings indicated that the highest percentage of MO dye removal was achieved under the conditions of pH 2, a temperature of 60 °C, agitation speed of 250 rpm, and adsorption duration of 90 min. Furthermore, the CS-ENH2-4 adsorbent exhibits a favorable potential for reusability, as it only experienced a reduction of 18% of its adsorption effectiveness after undergoing 10 cycles of adsorption–desorption. The structural, morphological, and physiochemical characterization of the developed amino-ethyl Chitosan derivatives underwent by Fourier Transform Infrared spectroscopy (FTIR), Thermal analysis (TGA and DSC), Scanning Electron Microscopy (SEM), and Energy Dispersive X-ray Analysis (EDAX). The later proved the amination process and the adsorption of MO through the increase of the N% by about 70%, in accordance with the results of the ion exchange capacity; 73%, and appearance of new S and Na elements in the analysis of MO-CS-ENH2-4, respectively.

## Data Availability

The datasets used and/or analysed during the current study available from the corresponding author on reasonable request.
